# Evaluating the diagnostic test accuracy of molecular xenomonitoring methods for characterising the community burden of Onchocerciasis

**DOI:** 10.1371/journal.pntd.0009812

**Published:** 2021-10-12

**Authors:** Joseph Pryce, Thomas R. Unnasch, Lisa J. Reimer

**Affiliations:** 1 Department of Vector Biology, Liverpool School of Tropical Medicine, Liverpool, United Kingdom; 2 Center for Global Health Infectious Disease Research, University of South Florida, Tampa, Florida, United States of America; National University of Ireland Galway, IRELAND

## Abstract

**Background:**

Molecular xenomonitoring (MX), the detection of parasite nucleic acid in the vector population, is recommended for onchocerciasis surveillance in elimination settings. However, the sensitivity of MX for detecting onchocerciasis-positive communities has not previously been evaluated. MX may have additional applications for control programmes but its utility is restricted by a limited understanding of the relationship between MX results and human prevalence.

**Methods:**

We conducted a systematic review of studies reporting the prevalence of *Onchocerca volvulus* DNA in wild-caught *Simulium* spp. flies (MX rate) and corresponding prevalence of microfilaria (mf) in humans. We evaluated the sensitivity of MX for detecting onchocerciasis-positive communities and describe the characteristics of studies with reduced sensitivity. We conducted a linear regression to evaluate the relationship between mf prevalence and MX rate.

**Results:**

We identified 15 relevant studies, with 13 studies comprising 34 study communities included in the quantitative analyses. Most communities were at advanced stages towards elimination and had no or extremely low human prevalence. MX detected positive flies in every study area with >1% mf prevalence, with the exception of one study conducted in the Venezuelan Amazonian focus. We identified a significant relationship between the two measurements, with mf prevalence accounting for half of the variation in MX rate (R^2^ 0.50, p<0.001).

**Conclusion:**

MX is sensitive to communities with ongoing onchocerciasis transmission. It has potential to predict human mf prevalence, but further data is required to understand this relationship, particularly from MX surveys conducted earlier in control programmes before transmission has been interrupted.

## Author summary

Traditional surveillance of onchocerciasis relies on the detection of *Onchocerca volvulus* microfilaria or antibodies in human skin or blood samples. Molecular xenomonitoring, the detection of parasite nucleic acid in vector insects, provides a non-invasive alternative. The sensitivity of molecular xenomonitoring to areas where infected people are found has not previously been evaluated and the extent to which xenomonitoring can be used to predict human prevalence is unknown. We searched for previous studies that reported the infection rates in humans and detection rates in black flies, finding 15 studies comprising 34 study communities that contributed to our analyses. Studies were conducted across Africa and the Americas, mostly in areas of very low prevalence. The findings show molecular xenomonitoring was sensitive to areas with greater than 1% microfilaria prevalence in the human population, indicating that molecular xenomonitoring is effective at detecting ongoing transmission. We further found evidence that infection rates in humans and detection rates in flies were related, providing scope for the use of xenomonitoring to predict human prevalence. With further research to better understand this relationship, control programmes may be able to use xenomonitoring for other purposes such as identifying areas that require intervention and monitoring the impact of treatments.

## Background

Onchocerciasis is a filarial disease associated with skin pathology and blindness [1]. Worldwide, 218 million people live in endemic areas, with more than 99% of those at risk living in Africa [2].

Mass drug administration (MDA) with the anthelminthic drug ivermectin is the primary method of controlling the disease. In the Americas, MDA programmes have helped to eliminate onchocerciasis from 11 of 13 previously endemic regions and the international community has outlined a MDA-based strategy for eliminating onchocerciasis from 80% of African countries by 2025 [3–5]. During onchocerciasis programmes, the treatment phase is conducted for 12–15 years, coinciding with the reproductive lifespan of adult worms. When onchocerciasis transmission has been successfully interrupted, the distribution of ivermectin is halted and programmes undertake 3–5 years of post-treatment surveillance followed by 3–5 years of post-elimination surveillance to ensure against resurgence of the disease [6].

### Surveillance of onchocerciasis

A variety of diagnostic methods have been used for the surveillance of onchocerciasis. The gold standard diagnostic technique involves superficial skin biopsies (known as ‘skin snips’) which are screened for *O*. *volvulus* microfilariae (mf) [7]. Communities with greater than 1% mf prevalence require treatment to interrupt transmission, but below this threshold transmission is considered to be unsustainable [8]. In communities with high infection intensity, ie. a high microfilarial load, skin snip surveys are both sensitive and specific. However, sensitivity is significantly reduced at low infection intensities [9]. As successful MDA implementation decreases both the mf prevalence and infection intensity within a community, the potential for false negatives increases as the programme progresses. In addition, skin snips are invasive and often painful, and programmes may encounter broad refusal from community members to participate in the surveys [10]. Consequently, skin snip surveys are not recommended for determining whether transmission has been interrupted or for post-treatment monitoring [6].

Serological methods are less invasive, requiring only a finger prick blood sample from participants. Control programmes often utilise an enzyme-linked immunosorbent assay (ELISA) to detect IgG4 antibodies to the *O*. *volvulus* antigen Ov16 [7]. Ov16 assays provide a more sensitive measure of exposure to *O*. *volvulus* parasites than parasitological surveys and consequently have greater use in the latter stages of elimination programmes [6]. However, Ov16 assays are unable to distinguish current infections from historical exposure [11]. Skin snips may therefore be needed to confirm whether Ov16 positive individuals have an active infection.

Molecular xenomonitoring (MX), the detection of parasite nucleic acid in vector insects, is a third surveillance option. The detection of *O*. *volvulus* DNA in the *Simulium* spp. black fly vectors of onchocerciasis can be used as a proxy indicator for parasite presence in the human population. The PCR-based diagnostic O-150 can detect *O*. *volvulus* DNA in pools of more than 100 flies, while an algorithmic tool can be used to reliably predict the proportion of vectors containing parasite DNA (hereafter defined as ‘MX rate’) from the pooled results [12,13]. Furthermore, the dissection and screening of black fly heads can be used to determine the proportion of flies containing infective stage larvae. A minimum of 6,000 black flies must be screened and confirmed negative to assure the upper bound of the 95% confidence interval for infectivity is below 0.05% (or 0.1% of parous flies, assuming a parity rate of 50%) and provide sufficient certainty that transmission has been interrupted [6]. MX overcomes many of the challenges associated with parasitological and serological surveillance as it measures current infections and does not require invasive sampling.

Currently, MX is recommended for onchocerciasis programmes aiming to demonstrate interruption or elimination of transmission [6]. MX may also become a valuable surveillance tool at earlier stages of control programmes, particularly if the results could be used to approximate the prevalence of onchocerciasis in the human population.

The development of the guidelines for onchocerciasis surveillance in elimination settings was supported by an unpublished review which based its conclusions primarily on the reports of two longitudinal observational studies [6]. In the years since the guidelines’ publication, several foci have eliminated onchocerciasis and new studies have been published that add to this data. In addition, the existing review did not provide a comparative analysis of MX against other surveillance methods. Consequently, the sensitivity of MX for identifying onchocerciasis-positive communities and the relationship between MX rates and mf prevalence is poorly understood.

### Aims and objectives

The overall aim of this review was to evaluate the diagnostic accuracy of MX methods for characterizing the community burden of onchocerciasis. The primary objective was to assess the sensitivity of MX for detecting onchocerciasis-positive areas and explore the factors that affect this sensitivity. A secondary objective was to evaluate the relationship between mf prevalence and MX rates, specifically exploring whether MX rates can predict whether mf prevalence is greater or lower than 1%.

## Methods

We conducted a systematic review and meta-analysis following the Preferred Reporting Items for Systematic Reviews and Meta Analyses guidelines [14]. The methods followed a protocol adapted from a recent review evaluating the accuracy of MX for lymphatic filariasis surveillance [15] and registered with the PROSPERO international database of prospectively registered systematic reviews in health and social care (CRD42021229511).

### Search strategy

We conducted an electronic search of five bibliographic databases incorporated into EBSCO host (CINAHL Complete, MEDLINE Complete, Global Health, eBook Collection, Global Health Archive) for records published up to 7^th^ January 2021. A complete description of the search terms is provided in S1 Table.

### Inclusion and exclusion criteria

Primary research articles were suitable for inclusion if they a) report the MX rate from wild-caught black flies and b) reported the human mf prevalence in the same area where black flies were collected.

For the reporting of MX rate, we placed no limitations on the species of black fly collected, methods used for black fly collection or molecular methods used for the detection of parasite genetic material. We included studies where MX methods were used to detect genetic material of any parasite life stage or infective parasites only, but we considered these outcomes to be distinct and analysed them separately. For the reporting of human mf prevalence, we included studies that collected and screened skin snip samples from the entire sampled population, as well as studies that used serological methods to detect individuals positive for antibodies and subsequently screened skin snips from each antibody-positive individual to confirm the mf prevalence in the overall sampled population. Accordingly, we also included studies that used serological methods and found zero antibody-positive cases, considering the mf prevalence to also be zero. We excluded studies where measurements of MX rate and mf prevalence were taken more than 18 months apart, or if MDA was distributed in the study area between the two time points.

### Selection of studies and data extraction

We screened the titles and abstracts of articles identified by the search and then screened the full texts of potentially relevant studies to identify those meeting the inclusion criteria. Using a prepared proforma, we extracted data on the geographical setting, study objectives, MDA history and methods used for sampling and screening of black fly and human populations. In the event of missing data, we made efforts to contact study authors for further information.

We extracted information at the smallest reported level, eg. individual villages within a district, if available. For each study area, we recorded the number of humans and black flies screened, measures of the biting density, mf prevalence, and MX rate. If data were presented graphically, we digitized the figures to obtain numerical values using the graph digitizing software WebPlotDigitizer (automeris.io/WebPlotDigitizer) and, where necessary, calculated the MX rate from the reported data using PoolScreen v2.0 [16].

### Assessment of methodological quality

We reviewed the methodological quality of included studies using an established tool for evaluating the quality of MX accuracy studies based on the QUADAS-2 tool [15,17]. The five criteria used to assess methodological quality were: whether the researchers interpreting the mf prevalence results were blinded to the MX rate results; whether the researchers interpreting the MX rate results were blinded to the mf prevalence results; whether there was a delay between MX and mf surveys; whether MX and mf surveys adequately targeted the same communities; and whether methods used were consistent across sampling timepoints. We further evaluated the applicability of human and black fly populations surveyed in each study in terms of their suitability for representing the overall human and black fly populations in the surveyed areas. For each criteria, studies were graded as high, low or unclear risk based on pre-determined specifications (S2 Table).

### Evaluation of sensitivity of MX

We evaluated the sensitivity of MX using standard methods for assessing diagnostic test accuracy adapted for the community-level detection of MX. We treated study areas as the unit of observation, and for each study area used binary measures of the presence/absence of mf-positive humans and presence/absence of black flies positive for *O*. *volvulus* DNA as the reference standard and index test results, respectively. For each study, we calculated the number of study sites that were true positives (+index; +reference), true negatives (-index; -reference), false positives (+index; -reference) and false negatives (-index; +reference). We calculated the sensitivity of MX as true positives / (true positives + false negatives). We plotted the sensitivity observed in each study in a Forest plot. As the dataset was too limited for our proposed analyses to determine an overall estimate of sensitivity and explore the factors affecting it, we provided a narrative summary of studies with reduced sensitivity.

### Evaluating the relationship between mf prevalence and MX rate

We conducted a linear regression to evaluate the relationship between mf prevalence and MX rate. We considered black fly biting density as a potential covariate and weighted the regression by black fly sample size. Forwards stepwise multiple linear regression methods were used to select the most suitable set of explanatory variables based on Akaike Information Criterion, using the stepAIC() function in the ‘MASS’ package of R version 3.6.2.

## Results

### Search results

**Fig 1:**
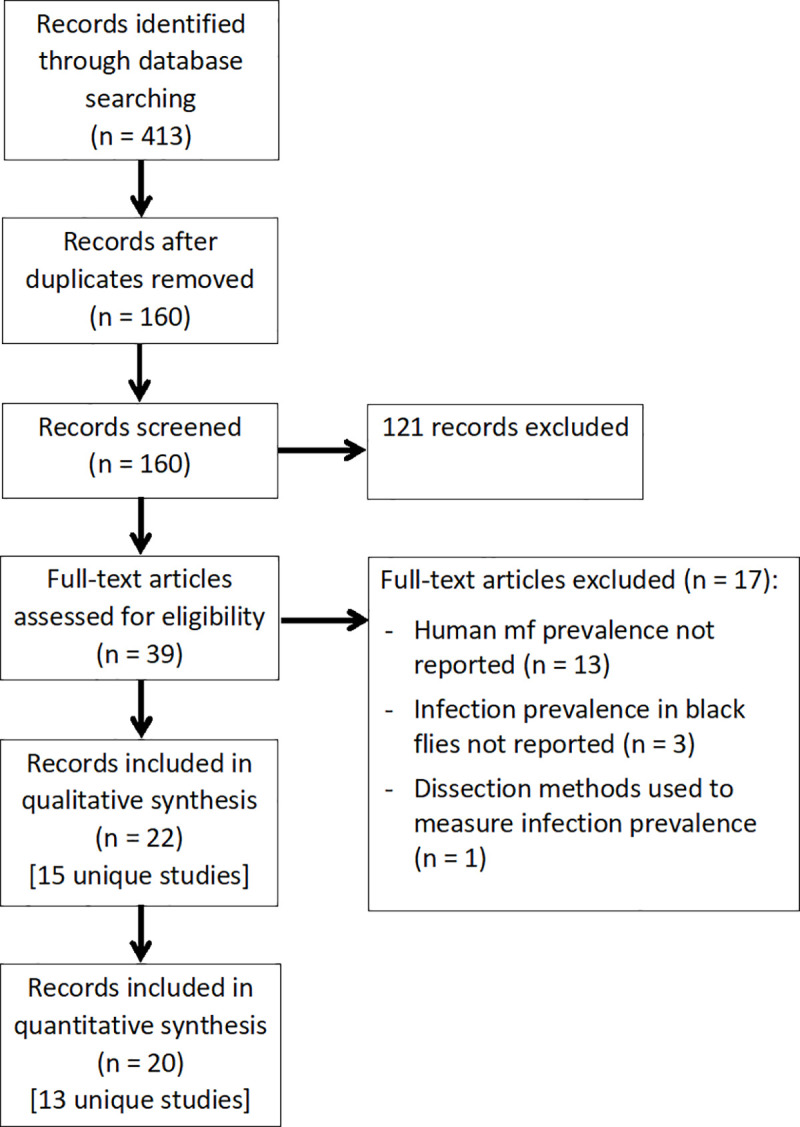
Flowchart showing the article selection process.

The electronic search strategy identified a total of 413 records. A total of 22 records corresponding to 15 unique studies met the inclusion criteria for the review [18–39]. Of these, 20 records corresponding to 13 unique studies were suitable for inclusion in the quantitative analysis (Fig 1).

### Characteristics of included studies

Included studies were conducted across a variety of geographical settings (Table 1). Almost all studies were conducted in areas in advanced stages of the elimination programme, with thirteen conducted with the objective of demonstrating transmission suppression or verifying elimination of transmission. None aimed to compare MX surveillance with epidemiological indicators.

**Table 1:** Characteristics of studies and study sites included in the reviews’ quantitative analyses. Abbreviations: mf–‘microfilaria’; pppts–‘per person per transmission season’; pppy–‘per person per year’.

Study [Reference]	Country	No. sites (timepoints)	No. humans screened	Human mf prevalence (%)	Black flies screened	Black fly pool size	Biting density
Botto 2016 [18]	Venezuela	3	Not stated	2.0 to 7.3%	27,666	200	13,048 to 130,143 bites pppts
Cruz-Ortiz 2012 [20]	Guatemala	1	3,118	0.0%	8,252	50^b^	5,765 bites pppts
Evans 2014 [22]	Nigeria	6	2,197	0.0 to 0.6%	1,568	100	700 bites pppts^a^
Guderian 1997 [23]	Ecuador	1 (2)	458	0.0 to 64%	20,000	50	10,710 bites pppts^a^
Katabarwa 2020a [27]	Sudan, Ethiopia	6	10,903	0.0%	74,891	200	Not reported
Katabarwa 2020b [26]	Uganda	1	2,953	0.0%	854	100	37 bites pppy
Komlan 2018 [28]	Togo	3	437	2.9 to 10%	4,475	25	15,519 bites pppy
Lindblade 2007 [29]	Guatemala	1	4,127	0.0%	11,621	50	2,380 bites pppts
Nicholls 2018 [30]	Colombia	1 (2)	375	0.0 to 0.85%	16,065	50^b^	2,919 to 73,958 bites pppts
Rodriguez-Perez 1999 [32,33]	Mexico	1	226	13.0%	10,550	50	Not reported
Rodriguez-Perez 2013 [34–36,39]	Mexico	4 (2); 2 (1)	>1,150	0.0 to 16%	>80,000	50	13,824 to 72,794 bites pppts
Traore 2012 [21,37]	Mali, Senegal	3 (2)	16,966	0.0 to 0.1%	492,600	300^b^	8,300 to 13,950 bites pppts^a^
Zarroug 2016 [24,25,38]	Sudan	1 (3)	6,244	0.0 to 0.5%	65,951	100	Not reported

^a^ Unpublished information estimated by reviewers from available study data

^b^ Unpublished information obtained through personal communication with study authors

A detailed report of each study’s survey methodology is presented in S3 Table. The included studies exclusively used human landing catches to collect black flies. Four studies screened pools of fly abdomens or whole carcasses and four studies screened pools of fly heads. Seven studies dissected fly heads from the bodies prior to DNA extraction and screened the bodies initially, with heads screened only if positive bodies were identified.

**Fig 2:**
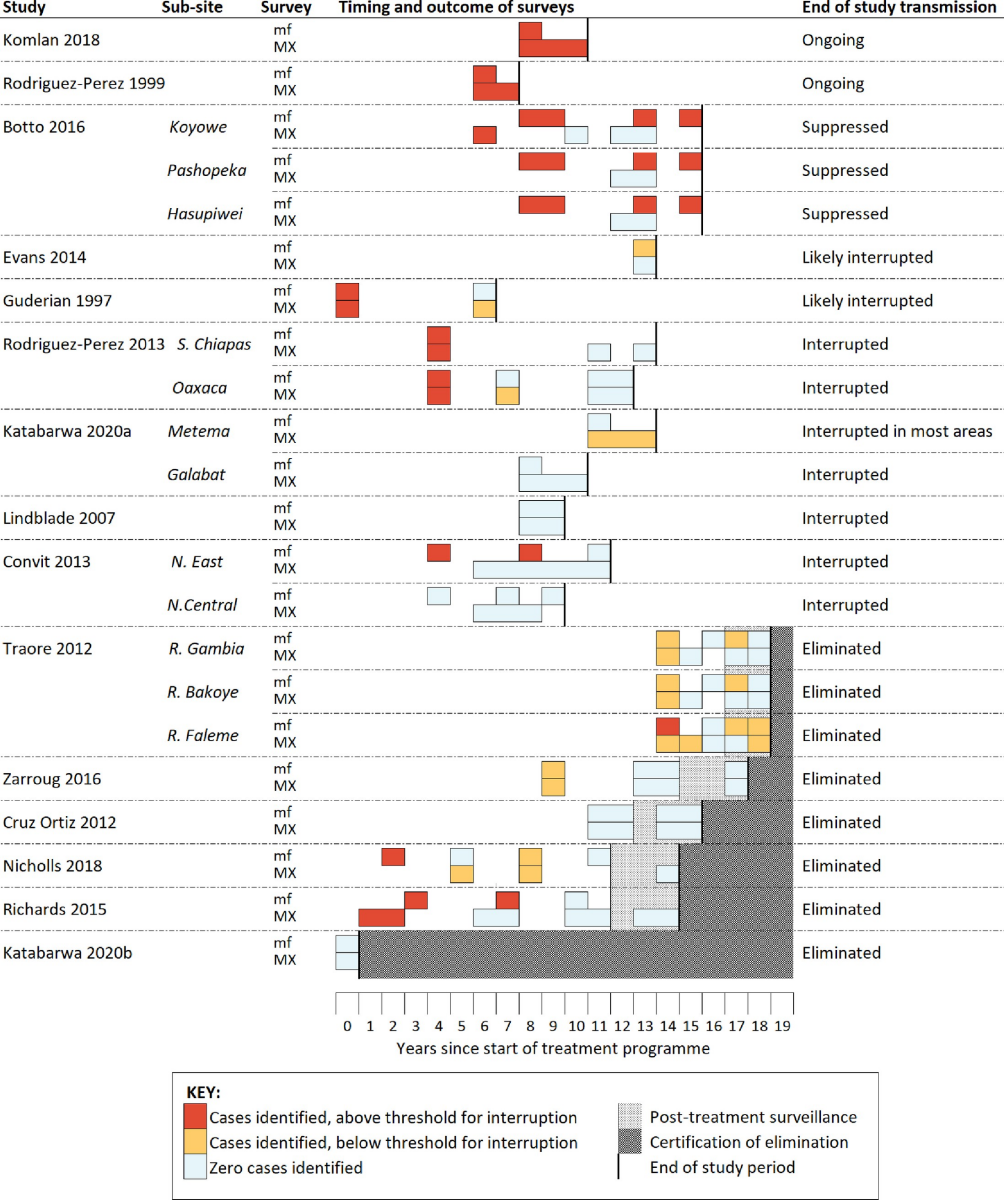
Summary of the timing and outcomes of entomological and parasitological surveys conducted during the time period of the included studies. Abbreviations: mf–‘microfilaria’; MX–‘molecular xenomonitoring’.

Across the 15 included studies, matched MX and mf survey data were available for 34 distinct areas, ranging in size from district to village level. The median number of people surveyed in each area was 352.5 (range 20 to 5,266). The median number of black flies screened per study area was 10,525 (range 110 to 122,100) with a median sampling effort of 1,210 hours per study area (range 77 to 8,032). A summary of the timing and outcomes of MX and epidemiological surveys that were conducted during each study is provided in Fig 2. Although many studies presented data collected using a variety of surveillance methods throughout the elimination process, only one study provided matched MX and mf data with a minimum of three sampling timepoints [38].

### Assessment of methodological quality

**Fig 3:**
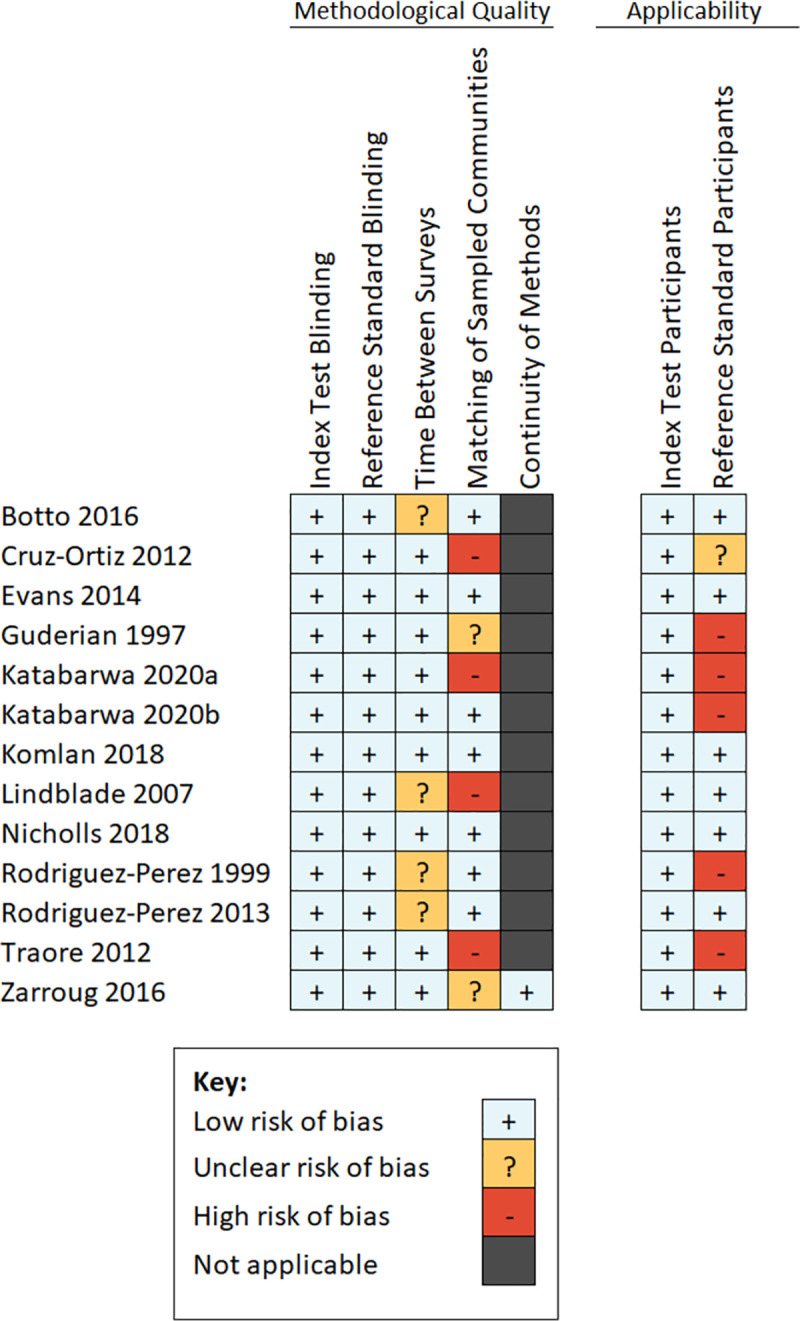
Summary of assessments of methodological quality.

Overall, there were few concerns about methodological quality across the included studies. In most studies, the mf and MX surveys were conducted within six months of one another. Two studies reported a combined result from MX surveys that were conducted over several years, during which time MDA had continued to be implemented biannually, and it was therefore not possible to extract paired mf and MX data from a single timepoint [19,31]. The data from these studies were therefore excluded from the review’s quantitative analyses. In four studies, the MX survey sampling points were limited in number and not matched to the specific villages in which mf surveys took place. Additionally, there were few concerns about applicability of the black fly populations screened by the included studies. Five studies limited their mf surveys to children and may therefore have reduced applicability to the overall human population. One study encountered challenges with participation in the last round of skin snip surveys with several entire villages refusing to take part. The absence of data from these villages was considered to reduce the applicability of the dataset. A summary of the quality assessments for each included study is provided in Fig 3. Details of the reasons given for each judgement are provided in S4 Table.

### Evaluating the sensitivity of MX

**Fig 4:**
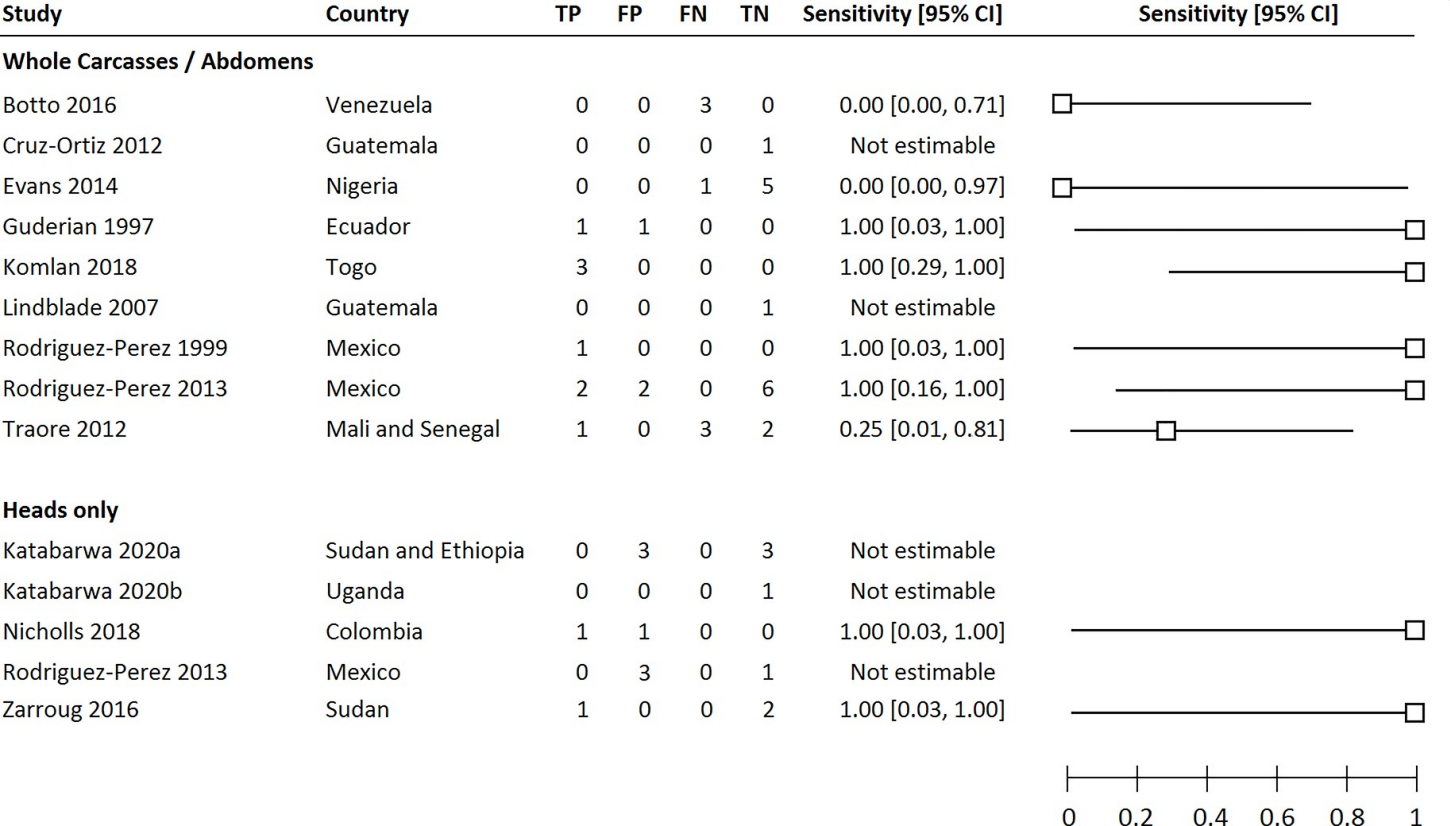
Forest plot summarising the sensitivity of MX of black flies, whether screening whole carcasses/abdomens or fly heads only, for the detection of communities that were positive for onchocerciasis as determined by human microfilaria prevalence surveys. Abbreviations: TP (True Positives), FP (False Positives), FN (False Negatives), TN (True Negatives).

Estimates of the sensitivity of MX for each of the included studies are shown in Fig 4. The quantitative data extracted from each included study is available in S5 Table.

Across the nine studies that screened whole carcasses or fly abdomens, mf-positive individuals were detected in 15 areas, of which positive flies were detected in eight (53.3%). Across the five studies that screened fly heads separately from abdomens, mf-positive individuals were identified in two study areas, of which positive heads were identified in both.

Overall, six studies detected positive flies in every study area in which mf-positive individuals were found [23,28,30,32,34,38]. In four studies, MX also detected positive flies in areas where no mf-positive individuals were detected [23,27,30,34]. However, there were three studies in which MX surveys found no positive flies despite the presence of mf-positive individuals [18,22,37]. In each case, the surveys were conducted in areas that had undergone a minimum of 12 years of MDA. In two of these studies, mf prevalence was below the threshold required to interrupt transmission; between 0.05 and 0.13% in Mali and Senegal [37], and 0.59% in Nigeria [22]. In the latter, the number of black flies sampled was also below the minimum recommendation of 6,000, with a total of 1,568 flies screened across six study areas. In the third study, the mf prevalence varied between 2.0 and 7.3% [18]. The study was conducted in the Amazon region of Venezuela, a challenging focus where the indigenous Yanomami population are highly migratory. Personal communication with the study authors suggested that this could have resulted in the detection of human cases and simultaneous collection of flies in areas other than where transmission occurred.

### Correlation between MX rate and mf prevalence

**Fig 5:**
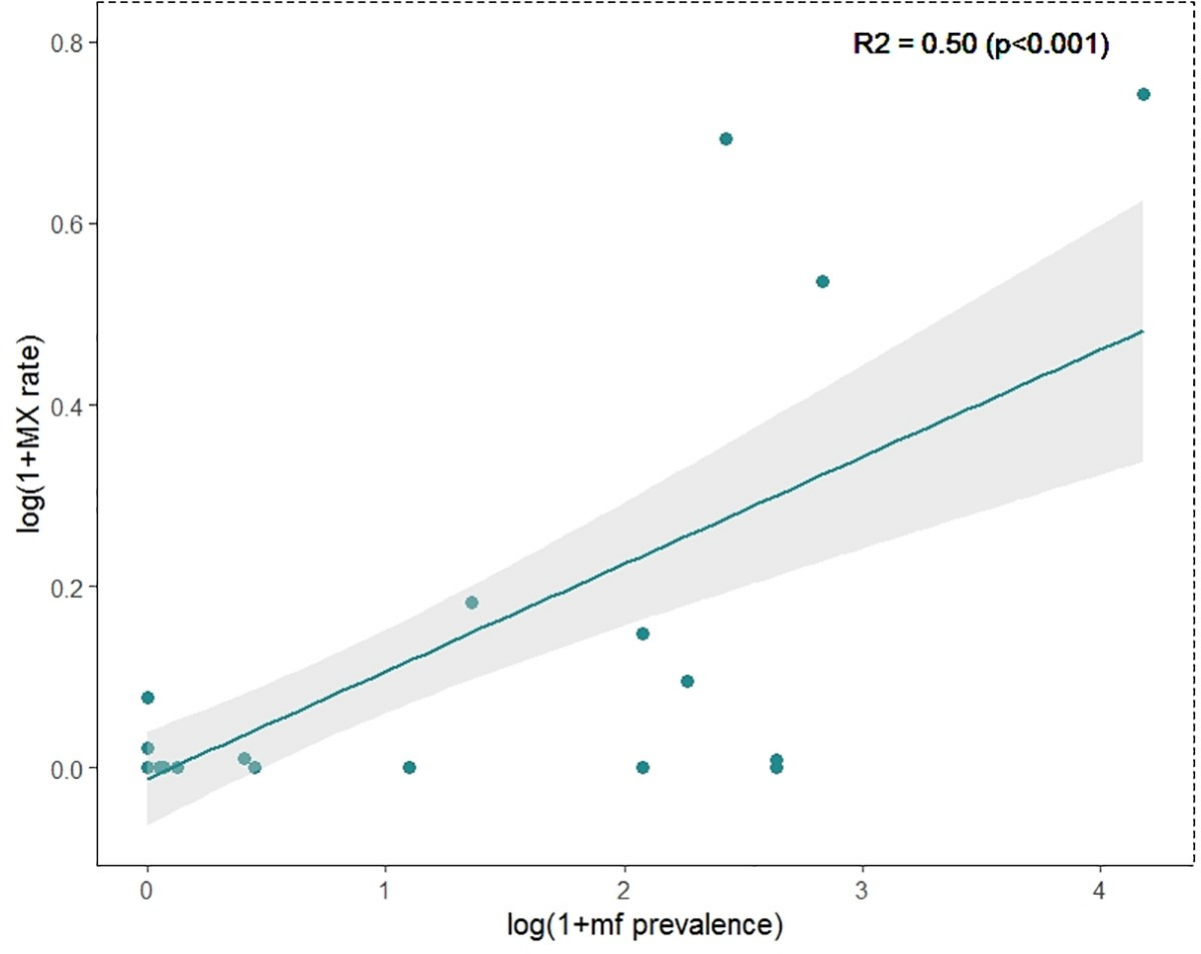
Linear regression model demonstrating the relationship between human microfilaria prevalence of onchocerciasis parasites and MX rate when screening black fly abdomens or whole carcasses.

Across the nine studies that screened fly abdomens or whole carcasses, mf prevalence was significantly associated with MX rate, though a large proportion of the variation between measurements of MX rate remained unexplained (R^2^ = 0.50, p < 0.001) (Fig 5). The inclusion of black fly biting density as a covariate did not improve the predictive power of the model.

Very few MX surveys were conducted in areas with mf prevalence near the threshold of 1%, preventing any analyses to determine the suitability of MX for predicting mf prevalence above or below this threshold. A total of six study areas reported a mf prevalence between zero and 1.0%, with four of these falling below 0.15%. No study areas reported a mf prevalence between 1.0 and 2.0%.

Data were insufficient to analyse the relationship between the MX rate in fly heads and mf prevalence in humans.

## Discussion

The utility of MX is currently restricted by an inability to interpret MX results in the context of the disease indicators that guide programme decisions. The aim of this review was to evaluate the suitability of MX for a range of programmatic goals.

With the exception of the Amazonian focus, MX detected positive flies in every study area in which the mf prevalence was greater than 1%. Given onchocerciasis transmission is considered unsustainable below this threshold, the reduced sensitivity of MX observed in communities with mf prevalence well below 1% may be operationally unimportant [8]. Furthermore, there were several study communities identified as onchocerciasis-positive by MX that were not detected by mf surveys. Such instances may indicate the detection of individuals with potentially high mf levels that do not participate in MDA programmes or skin snipping surveys and otherwise present a challenge to control programs. Overall, this evidence exhibits the strengths of MX in identifying areas of ongoing transmission and supports the existing recommendations for MX use to determine whether transmission has been interrupted and for post-treatment surveillance [6]. However, these conclusions are drawn from an extremely limited data set where very few study areas were mf positive, preventing a precise estimate of the sensitivity of MX.

The failure to detect positive flies in the Amazonian focus may be due to the migratory behaviour of the human population, which makes the comparison of entomological and epidemiological surveys in the region difficult. However, there could be several potential explanations for observing unexpected MX results. MX sensitivity may be affected by the sample size or species composition of collected black flies, with different species exhibiting distinct ecological, behavioral, and vectorial capacity traits. Unfortunately, we were unable to quantitatively evaluate the degree to which these variables influenced sensitivity.

Our secondary analysis shows evidence of a linear relationship between MX rate and mf prevalence. These findings demonstrate potential of MX to aid control programmes at earlier stages of progress towards elimination, for example when monitoring the impact of treatment, without conducting invasive surveys. Half of the observed variation in MX rate across the studies was explained by variation in human mf prevalence, indicating a correlation between mf and MX rates similar to that seen for lymphatic filariasis [15]. The remaining variation may be explained by differences in geographical setting and sampling methods between the included studies. While evidence from lymphatic filariasis studies suggests that a greater correlation will be observed in a given setting with consistent sampling methodology [15], there was a lack of data from longitudinal studies to validate this observation for onchocerciasis. In addition, we were unable to evaluate the accuracy of MX for determining whether a community mf prevalence is greater or lower than 1% due to the absence of data from areas at or near this threshold.

Data comparing MX surveys with concurrent parasitological surveys across a greater range of transmission levels would strengthen our certainty in the existing recommendations and help inform our interpretation of MX results for other programmatic goals. Collection of such data could be facilitated by control programmes utilizing MX methods earlier in the elimination pathway. Other areas for future research include improving the sustainability of MX, for example through the development of a sampling strategy that does not depend on human landing catches. Though this has been the primary method for sampling black flies for decades, concerns over the time-intensiveness, inefficiency and, in particular, the ethical suitability of such methods have led to calls for novel methods for sampling black fly vectors to be developed [40]. As each of the studies included in this review depended on human landing catches, we are currently unable to say with certainty whether MX would still be expected to detect onchocerciasis-positive communities if alternative vector trapping methods were used.

## Conclusions and recommendations

The sensitivity of MX in areas of ongoing onchocerciasis transmission supports the current recommendations for MX use in elimination settings. While a large degree of variation in MX rates could not be explained by mf prevalence alone, evidence of a relationship between the two variables provides scope for expanding the utility of MX in future to include predicting the prevalence of disease and monitoring the impact of treatment. To improve our understanding of the potential of MX to meet these programmatic goals, valuable information will come from control programmes that utilise MX methods earlier in their progress before transmission has been interrupted.

## Supporting information

S1 Table Search terms and search strategy used to retrieve articles relating to the use of MX methods for onchocercerciasis surveillance. (DOCX)

S2 Table Assessment criteria and marking strategy for evaluating methodological quality. (DOCX)

S3 Table Characteristics of included studies. (DOCX)

S4 Table Explanations for assessments of methodological quality. (DOCX)

S5 Table Quantitative data extracted from included Studies. (DOCX)
