# Supplementary material for: Evaluating the diagnostic test accuracy of molecular xenomonitoring methods for characterising the community burden of Onchocerciasis
Source: PLoS Negl Trop Dis. 2021 Oct 12;15(10):e0009812. doi: 10.1371/journal.pntd.0009812 (PMC8509893; doi:10.1371/journal.pntd.0009812)
Supplement: S1 Table — (DOCX) [file pntd.0009812.s001.docx]

**S1 Table: Search terms and search strategy used to retrieve articles relating to the use of MX methods for onchocercerciasis surveillance**

| **Search steps** | **Search terminology** |
| --- | --- |
| #1 | Xenosurveillance OR Xeno-surveillance |
| #2 | Xenomonitor* OR Xeno-monitor* |
| #3 | Blackfl* OR Simulium OR “Black fl*” |
| #4 | “Molecular screen*” OR “Molecular diagnos*” OR PCR OR “Polymerase chain reaction” OR DNA |
| #5 | #3 AND #4 |
| #6 | #1 OR #2 OR #5 |
| #7 | Onchocerc* OR “River blindness” |
| #8 | #6 AND #7 |
