# Supplementary material for: Evaluating the diagnostic test accuracy of molecular xenomonitoring methods for characterising the community burden of Onchocerciasis
Source: PLoS Negl Trop Dis. 2021 Oct 12;15(10):e0009812. doi: 10.1371/journal.pntd.0009812 (PMC8509893; doi:10.1371/journal.pntd.0009812)
Supplement: S2 Table — (DOCX) [file pntd.0009812.s002.docx]

**S2 Table: Assessment criteria and marking strategy for evaluating methodological quality**

| **Domain** | **Description** |
| --- | --- |
| Blinding of Index Test | *Were the index test results interpreted without knowledge of the results of the reference standard?*  This item is similar to “blinding” in intervention studies. Interpretation of index test results may be influenced by knowledge of the reference standard. The potential for bias is related to the subjectivity of index test interpretation and the order of testing.   If the index test was always conducted and interpreted prior to the reference standard, this item was rated “low risk”. If it was not described whether the outcomes of the reference standard were known to those conducting the index test, the study was typically considered to have an “unclear risk”. However, if the objective of the study was not to evaluate the use of the index test, such cases were also considered to be “low risk”. |
| Blinding of Reference Test | *Were the reference standard results interpreted without knowledge of the results of the index test?*  This item is similar to the signalling question related to interpretation of the index test. Potential for bias is related to the potential influence of prior knowledge on the interpretation of the reference standard.   As above, if it was not described whether the outcomes of the index test were known to those conducting the reference standard, the study was typically considered to have an “unclear risk”. However, if the objective of the study was not to evaluate the use of the index test, such cases were also considered to be “low risk”. |
| Length of Time Between Surveys | *Was the interval between index test and reference standard appropriate?*   Ideally, index test and reference standard surveys would be collected in the same communities at the same time. If there is a significant delay, or if MDA has been implemented between the index test and reference standard, misclassification may occur due to an increase or decrease in disease prevalence between the two surveys.  We considered a gap of less than 6 months between to be “low” risk. For gaps of more than 6 months and less than 12 months, or if the length of time between surveys was not described, we considered the risk to be “unclear”. For gaps of more than 12 months but less than 18 months the risk was considered “high”. If the gap was more than 18 months, or if MDA had been implemented in between, the comparison was considered unsuitable for inclusion in the review. |
| Matching of Sampled Communities | *Were the communities in which the index test and reference standards were conducted appropriately matched?*  Ideally, the index test and reference standard surveys would be conducted in exactly the same areas. If the surveyed area is large, eg. district level, with MX surveys conducted in one village and mf surveys conducted in a distant village, the surveys may not be expected to provide comparable results.   We considered surveys that were undertaken in the same villages to be “low” risk, even if the households targeted for entomological and parasitological surveys were not identical. However, if surveys were conducted at a district or sub-district level and MX surveys were carried out in specific communities within the district or sub-district that was not matched by mf surveys, we considered this to be “high” risk. If there was not information about sampling strategies to form a judgement, studies were graded as “unclear” risk |
| Continuity of Methods  [Longitudinal studies only] | *Could variation in methodology used at different time points have introduced bias?*  Any variation in the methodology used for the sampling of locations, the index test or reference standard may lead to systematic changes in the outcome data collected.   We considered studies to be at “low” risk if there was no or limited variation in methodology, and “high” risk if there was significant variation that we felt could influence measurements of MX rate and mf prevalence. If there was not information about sampling strategies to form a judgement, studies were graded as “unclear” risk. |
| Index test participant applicability | *Do the index test participants match the review question?*  We considered studies to be at “high” risk if we had concerns that the sampling strategy targeted a limited population that was not widely applicable to the general black fly population in the area. We considered studies to be at “low” risk if we had no such concerns, and “unclear” if there was not enough information to make a decision. |
| Reference standard participant applicability | *Do the reference standard participants match the review question?*  We considered studies to be at “high” risk if we had concerns that the sampling strategy targeted a limited population that was not widely applicable to the general human population in the area. We considered studies to be at “low” risk if we had no such concerns, and “unclear” if there was not enough information to make a decision. |
