# Supplementary material for: Evaluating the diagnostic test accuracy of molecular xenomonitoring methods for characterising the community burden of Onchocerciasis
Source: PLoS Negl Trop Dis. 2021 Oct 12;15(10):e0009812. doi: 10.1371/journal.pntd.0009812 (PMC8509893; doi:10.1371/journal.pntd.0009812)
Supplement: S3 Table — (DOCX) [file pntd.0009812.s003.docx]

**S3 Table: Characteristics of included studies**

| **Botto 2016** | |
| --- | --- |
| **Study Location** | Venezeula. South, Amazonian region |
| **Timing of study** | Final surveys conducted in 2015 with data reported previous 20 years.  Relevant data for review collected in 2012-13 |
| **Epidemiological background** | Combination of hypoendemic, mesoendemic and hyperendemic communities prior to treatment. Sentinel communities were typically hyperendemic. |
| **History of MDA** | 6 monthly ivermectin treatment began in 2000. >85% coverage sustained since In 2009, treatment frequency began to increasee to quarterly regimens and at the time of the study had been extended to 192 out of 241 (80 %) of the endemic communities in the focus. |
| **History of other interventions** | None described. |
| **Study design** | Longitudinal, with data presented from baseline and follows 15 years of MDA. Only data from 2013 from 3 villages is suitable for inclusion in the review analyses |
| **Study objectives** | To find evidence of transmission suppression in the region after 15 years of MDA |
| **Sampling strategy (reference standard)** | 16 sentinel / extra-sentinel communities were selected for ongoing parasitological monitoring (including the four which were selected for entomological monitoring). |
| **Frequency and timing of sampling (reference standard)** | Direct observation of skin snips (2001, 2008, 2013, 2015). Opthalmological evaluations to detect presence of mf in the cornea or anterior chamber (2001, 2008, 2015). |
| **Oncho diagnostic method** | Direct observation of skin snips and opthalmological evaluations to detect presence of mf in the cornea or anterior chamber |
| **Sampling strategy (index test)** | Three sentinel (Hasupiwei, Pashopëka, Koyowë) and one extra-sentinel (Arokofita) community |
| **Frequency and timing of black fly sampling** | 3-12 consecutive days in each community, during the high transmission seasons (Jan-April and Sep-Nov). The number of collection days depended on the biting density in each community in order to reach a number of at least 6000 flies. Surveys were conducted in each of the four vilages in 2012-13. One community was additionally surveyed in 2006 and 2010. |
| **Black fly trapping method** | HLC. 2 collectors and two attractants working for approximately 7 hours per day until target sample was reached. |
| **Sample type (heads / bodies / whole carcasses etc)** | Body pools were analyzed first; if any of those pools were positive, all of the head pools analyzed to provide an estimate of the infectivity rate |
| **Max pool size** | 200 |
| **Convit 2013** | |
| **Study Location** | Venezuela. North Central and North East foci |
| **Timing of study** | Final surveys conducted in 2012 with data reported previous 12 years.  No matched data suitable for review |
| **Epidemiological background** | Combination of hypoendemic, mesoendemic and hyperendemic communities prior to treatment. Sentinel communities were typically hyperendemic. |
| **History of MDA** | 6 monthly ivermectin treatment began in 2001. >85% coverage sustained since In 2004/5. |
| **History of other interventions** | None described. |
| **Study design** | Longitudinal, with data presented from baseline prior and following 12 years of MDA. |
| **Study objectives** | To find evidence of transmission suppression in the region after 12 years of MDA |
| **Sampling strategy (reference standard)** | NC: 1 sentinel and 4 extra-sentinel communities (<174 inhabitants per community).  NE: 5 sentinel and 8 extra-sentinel communities (<206 inhabitants per community). |
| **Frequency and timing of sampling (reference standard)** | North Central: 4 parasitological (2001, 2005, 2008, 2010) and 2 ophthalmologic (2008, 2010) surveys were carried out in the sentinel community. North East: 4 parasitological (2001, 2006, 2009, 2012) and 4 ophthalmologic (2001, 2006, 2009, 2012) surveys |
| **Oncho diagnostic method** | Direct observation of skin snips and opthalmological evaluations to detect presence of mf in the cornea or anterior chamber |
| **Sampling strategy (index test)** | NC: 1 sentinel and 4 extra-sentinel communities (<174 inhabitants per community).  NE: 5 sentinel and 8 extra-sentinel communities (<206 inhabitants per community). |
| **Frequency and timing of black fly sampling** | North Central: September 2007 to March 2008 in the sentinel community, and September to November 2009 in the extra-sentinel communities.   North-east:  September 2007 to March 2008 and during 2011 in the 5 sentinel communities. September to November 2010 and 2012 in 5 extra-sentinel communities. |
| **Black fly trapping method** | HLC. 2 collectors and two attractants working for approximately 8 hours per day until target sample was reached. |
| **Sample type (heads / bodies / whole carcasses etc)** | Body pools were analyzed first; if any of those pools were positive, all of the head pools analyzed to provide an estimate of the infectivity rate |
| **Max pool size** | 50 |
| **Cruz-Ortiz 2012** | |
| **Study Location** | Guatemala. Huehuetenango Focus |
| **Timing of study** | 2007-8 |
| **Epidemiological background** | The area was showing a gradual reduction in infection prevalence before the initiation of MDA. Skin snip mf prevalence < 1% in 1992 |
| **History of MDA** | Annual Ivermectin treatment began in 1996. Increased to twice annually in 2000. >85% coverage sustained since In 2002.. |
| **History of other interventions** | None described. |
| **Study design** | Cross-sectional |
| **Study objectives** | To find evidence of transmission suppression in the region after 12 years of MDA |
| **Sampling strategy (reference standard)** | For skin mf surveys, children age 6-12 were surveyed by randomly selecting schools in the area until a target of 3000 children was reached. For opthalmological examinations, 40 residents (13 houses) randomly sampled from each of nine communities, which were selected from among the 19 eligible communities. |
| **Frequency and timing of sampling (reference standard)** | September 2007 |
| **Oncho diagnostic method** | Serology of school children to detect IgG antibodies to OV16 antigen (no positives detected) and opthalmological evaluations to detect presence of mf in the cornea or anterior chamber |
| **Sampling strategy (index test)** | Samples were collected in four coffee plantations located in the same geographical areas of the potentially endemic communities. |
| **Frequency and timing of black fly sampling** | Twice per month from November 2007 to April 2008. |
| **Black fly trapping method** | HLC. Four attractants and Four collectors at each of four plantations, working for approximately two days per month. |
| **Sample type (heads / bodies / whole carcasses etc)** | Not described. |
| **Max pool size** | 50 |

| **Evans 2014** | |
| --- | --- |
| **Study Location** | Nigeria.Central, Platea and Nasarawa |
| **Timing of study** | 2009 |
| **Epidemiological background** | Baseline skin mf prevalence of 42.95% |
| **History of MDA** | Annual MDA with ivermectin began in 1992, in combination with albendazole since 2001. |
| **History of other interventions** | None described |
| **Study design** | Cross-sectional |
| **Study objectives** | To find evidence of transmission suppression in the region after 17 years of MDA |
| **Sampling strategy (reference standard)** | 6 sentinel villages. All eligible residents of the six sentinel villages were invited to participate and all those who arrived on the appointed day were enrolled. |
| **Frequency and timing of sampling (reference standard)** | 2009, 12–14 months after the last round of MDA |
| **Oncho diagnostic method** | direct observation of snip snips for mf |
| **Sampling strategy (index test)** | Vector breeding sites near each of the 6 sentinel villages |
| **Frequency and timing of black fly sampling** | June-August 2009 |
| **Black fly trapping method** | HLC. Teams of four at each sentinel site, four days per month for 11 hours per day |
| **Sample type (heads / bodies / whole carcasses etc)** | Body pools were analyzed first; if any of those pools were positive, all of the head pools analyzed to provide an estimate of the infectivity rate |
| **Max pool size** | 100 |

| **Guderian 1997** | |
| --- | --- |
| **Study Location** | Ecuador. Rio Santiago focus |
| **Timing of study** | January 1990 to December 1996 |
| **Epidemiological background** | Combination of hypoendemic, mesoendemic and hyperendemic communities prior to treatment. Sentinel communities were hyperendemic. |
| **History of MDA** | Ivermection MDA started in 1990 and continued until 1996. 6 monthly in hyperendemic communities, annually in other. |
| **History of other interventions** | None described. |
| **Study design** | Longitudinal |
| **Study objectives** | To report the impact of 7 years of MDA on disease transmission |
| **Sampling strategy (reference standard)** | All children under 5 in hyperendemic communities were sampled for skin snip survey. |
| **Frequency and timing of sampling (reference standard)** | Skin snips were taken every 6 months, from 1990 to 1996 from a cohort of infected participants. Skin snips were taken from all children <5 in 1990 priort to ivermectin and 1996 only. Opthalmological examinations were also conducted in 1990 and 1995. |
| **Oncho diagnostic method** | Direct observation of skin snips and opthalmological evaluations to detect presence of mf in the cornea or anterior chamber.  Only the skin snip survey is conducted within a suitable time frame for comparison with the entomological survey |
| **Sampling strategy (index test)** | Not described. Targeted 10,000 black flies |
| **Frequency and timing of black fly sampling** | April and May in 1989 and 1996. |
| **Black fly trapping method** | HLC. Over a 7-day period, 10000 black flies were captured by 8 volunteers, between 6 a.m. and 5 p.m. |
| **Sample type (heads / bodies / whole carcasses etc)** | Not described |
| **Max pool size** | 50 |

| **Katabarwa 2020a** | |
| --- | --- |
| **Study Location** | Sudan and Ethiopia, Galabat-Metema focus. |
| **Timing of study** | 2014 - 2017 |
| **Epidemiological background** | Onchocercal disease was reported to be a major problemin the districts. Skin mf prevalence in Galabat focus was reported as 52.5% |
| **History of MDA** | Once per year MDA began in Ethiopia's Metema focus in 2003 and the Sudan's Galabat focus in 2007. Twice per year MDA began in Ethiopia's Metema focus in 2016 and the Sudan's Galabat focus in 2008. |
| **History of other interventions** | None-described |
| **Study design** | Cross-sectional |
| **Study objectives** | To see if either subfocus met the requirements for stopping MDA. |
| **Sampling strategy (reference standard)** | Sampling in each subfocus was conducted to get a minimum of 3000 children aged five to 10 years of age. Villages were selected based on known history of onchocerciasis and closeness to the border.  Surveys were also conducted in adjacent districts Alefa, Quara, Tach Armachicho and Chilga. In each village, 100 children (5 to 10 years) were included. |
| **Frequency and timing of sampling (reference standard)** | One survey in each district, occuring between 2015 and 2017. |
| **Oncho diagnostic method** | OV16 ELISA testing of dried blood samples. All OV-16 positive children were followed up for skin snip collections which were screened using o-150 PCR. |
| **Sampling strategy (index test)** | Targeted at least 6000 vectors per focus. 10 collection sites were identified (7 in Metema and 3 in Galabat).   Surveys were also conducted in 6 fly collection sites in the adjacent districts Alefa, Quara, Tach Armachicho and Chilga. |
| **Frequency and timing of black fly sampling** | June 2014 to February 2015 (study areas).  November 2015 to November 2016 in adjacent districts.  Monthly. |
| **Black fly trapping method** | HLC. Collections conducted 11 hours a day, 10 days per month for 9 months. |
| **Sample type (heads / bodies / whole carcasses etc)** | Heads |
| **Max pool size** | 200 |

| **Katabarwa 2020b** | |
| --- | --- |
| **Study Location** | Uganda, Victoria Nile focus |
| **Timing of study** | Feb 2017 - Feb 2018 |
| **Epidemiological background** | The focus was considered to have eliminated onchocerciasis since 1967 after elimination of the vector species S. damnosum. However this had not been confirmed by recent WHO standards |
| **History of MDA** | None described |
| **History of other interventions** | Historical elimination of the vector species Simulium damnosum sl using insecticide air sprays, drips and larviciding |
| **Study design** | Cross-sectional |
| **Study objectives** | To verify elimination of onchocerciasis in the focus by WHO guidelines. |
| **Sampling strategy (reference standard)** | Villages at highest risk of bites from Simulium (those located nearest to the river) were surveyed |
| **Frequency and timing of sampling (reference standard)** | One survey in 2017 |
| **Oncho diagnostic method** | OV16 ELISA testing of dried blood samples. All samples were OV16 negative |
| **Sampling strategy (index test)** | Targeted 6 historical vector breeding sites and one additional potential breeding site along the river. |
| **Frequency and timing of black fly sampling** | Feb 2017 - Feb 2018 |
| **Black fly trapping method** | HLC. Two fly collectos at each collection site, 11 hours a day, 2 days per week (8 days per site per month). |
| **Sample type (heads / bodies / whole carcasses etc)** | Heads.  Note, only S. adersi flies were identified, which is not a known vector of O. volvulus |
| **Max pool size** | 100 |

| **Komlan 2018** | |
| --- | --- |
| **Study Location** | Togo, North and Central regions. River basins of Oti, Keran and Mo. |
| **Timing of study** | 2015 - 2017 |
| **Epidemiological background** | Following vector control and MDA implementation, epidemiological surveys conducted by the national conttol programme have shown mf prevalence below 5% (and below 1% in children under 10) in most hyperendemic districts. |
| **History of MDA** | MDA with ivermectin implemented since 1988. Biannual ivermectin implemeted in special transmission areas from 2007 until 2012. |
| **History of other interventions** | Vector control from 1987 until 2007 |
| **Study design** | Cross-sectional |
| **Study objectives** | To assess the current epidemiological situation and to determine whether transmission has been interrupted and ivermectin MDA can be stopped. |
| **Sampling strategy (reference standard)** | For this study, 11 sentinel villages were selected. 3 were matched with fly collection villages |
| **Frequency and timing of sampling (reference standard)** | One survey - timing not stated |
| **Oncho diagnostic method** | Direct observation of skin snips |
| **Sampling strategy (index test)** | The collection of S. damnosum s.l. was conducted at 4 specific catch points at river sites in proximity to sentinel villages in the Oˆti river basin (village Pance´rys/ Savanes Region), Kara (village Tchitchira/Kara Region), Moˆ (village Baghan/Kara Region and Bouzalo/Central Region). 3 were matched with parasitological survey sites. |
| **Frequency and timing of black fly sampling** | Aug-Sep 2015 and until 2017 in the Mo study site |
| **Black fly trapping method** | HLC. 11 hours a day for 5 days a week |
| **Sample type (heads / bodies / whole carcasses etc)** | Whole flies |
| **Max pool size** | 25 |

| **Lindblade 2007** | |
| --- | --- |
| **Study Location** | Guatemala, Santa Rosa focus |
| **Timing of study** | 2004-2005 |
| **Epidemiological background** | Hypoendemic |
| **History of MDA** | MDA with ivermectin implemented since 1996 and high coverage of biannual treatment sustained from 2000 |
| **History of other interventions** | None described |
| **Study design** | Cross-sectional |
| **Study objectives** | To verify elimination of onchocerciasis in the focus by WHO guidelines. |
| **Sampling strategy (reference standard)** | Serological survey: All schoolchildren 6–12 years of age in the 43 schools serving the 70 potentially endemic communities and a 2 km buffer around each PEC to reach a target of 3000 children  Opthalmological survey: 6 of 70 potentially endemic communities were selected based on history of nodule prevalence >0% in last 3 surveys and elevatino > 800 m. All community members > 7 years old were asked to participate. |
| **Frequency and timing of sampling (reference standard)** | Not described |
| **Oncho diagnostic method** | Serology of school children to detect IgG antibodies to OV16 antigen (no positives detected) and opthalmological evaluations to detect presence of mf in the cornea or anterior chamber |
| **Sampling strategy (index test)** | 8 collection sites in 7 of the 70 potentially endemic communities were selected based on high vector density and willingness to participate. |
| **Frequency and timing of black fly sampling** | Twice monthly between Dec 2004 and Apr 2005. |
| **Black fly trapping method** | HLC. Four teams of one collector and one attractant working for 8 hours per day |
| **Sample type (heads / bodies / whole carcasses etc)** | Body pools were analyzed first; if any of those pools were positive, all of the head pools analyzed to provide an estimate of the infectivity rate |
| **Max pool size** | 50 |

| **Nicholls 2018** | |
| --- | --- |
| **Study Location** | Colombia. Naicioná village |
| **Timing of study** | 1998 - 2010 |
| **Epidemiological background** | Pre-treatment skin snip mf prevalence of 40% |
| **History of MDA** | 12 years of biannual ivermection MDA from 1996 with 85% coverage achieved from 1999. |
| **History of other interventions** | None described. |
| **Study design** | Longitudinal, however only surveys conducted in 2001 and 2004 provide matched data of parasitological and entomological data |
| **Study objectives** | To verify elimination of onchocerciasis in the focus by WHO guidelines. |
| **Sampling strategy (reference standard)** | Not described |
| **Frequency and timing of sampling (reference standard)** | Relevant surveys in 2001 and 2004 |
| **Oncho diagnostic method** | Skin snip survey. (confirmation of mf method not described) |
| **Sampling strategy (index test)** | Blackfly collections were done at known transmission sites in the community of Naicioná. Several sampling sites were selected. |
| **Frequency and timing of black fly sampling** | Collections were carried out once a month, between July and December. |
| **Black fly trapping method** | Not described |
| **Sample type (heads / bodies / whole carcasses etc)** | Heads |
| **Max pool size** | 50 |

| **Richards 2015** | |
| --- | --- |
| **Study Location** | Guatemala, Central Endemic Zone |
| **Timing of study** | 2003-2014 |
| **Epidemiological background** | Baseline skin mf prevalence of ~70% |
| **History of MDA** | MDA with ivermectin implemented since 1988 and high coverage of biannual treatment sustained from 2000 to 2011. |
| **History of other interventions** | None described. |
| **Study design** | Longitudinal with multiple surveys conducted during the MDA period. Matched data is provided from 2007 and 2010-2011. |
| **Study objectives** | Report of recent surveys leading to the declaration that transmission had been eliminated in the zone |
| **Sampling strategy (reference standard)** | 9 sentinel villages were selected for the area, SVs were selected from among the most highly endemic communities for onchocerciasis |
| **Frequency and timing of sampling (reference standard)** | Matched data is provided from 2007 and 2010-2011. |
| **Oncho diagnostic method** | Direct observation of superficial skin biopsies, obtained from those who were ≥ 5 years old. Slit lamp examinations were conducted by an experienced ophthalmologist in residents ≥ 7 years old |
| **Sampling strategy (index test)** | 4 |
| **Frequency and timing of black fly sampling** | Matched data is provided from 2007 and 2010-2011.  Surveys conducted from November - April. |
| **Black fly trapping method** | HLC. One collector and one attractant working 8 hours per day |
| **Sample type (heads / bodies / whole carcasses etc)** | Body pools were analyzed first; if any of those pools were positive, all of the head pools analyzed to provide an estimate of the infectivity rate |
| **Max pool size** | 50 |

| **Rodriguez-Perez 1999** | |
| --- | --- |
| **Study Location** | Mexico. Las Golondrinas, southern Chiapas focus |
| **Timing of study** | 1997 - 1998 |
| **Epidemiological background** | Formerly hyper-endemic (78% skin mf prevalence) |
| **History of MDA** | 7 years (11 rounds) of ivermectin MDA |
| **History of other interventions** | 7 years (11 rounds) of nodulectomy |
| **Study design** | Cross-sectional entomological survey presented alongside longitudinal monitoring of human infection prevalence.  Only 1997 parasitological data provides a comparison |
| **Study objectives** | To compare a PCR assay for the detection of O. volvulus DNA with dissection-based methods |
| **Sampling strategy (reference standard)** | All children under 18 with consenting parents |
| **Frequency and timing of sampling (reference standard)** | Annually from 1994 - 1998 (only 1997 provides a comparison with entomological data+J12 |
| **Oncho diagnostic method** | Direct observation of skin snips |
| **Sampling strategy (index test)** | 4 collection sites - 2 within the village and 2 in a nearby coffee plantation of Las Golondrinas |
| **Frequency and timing of black fly sampling** | April 1997 - February 1998 |
| **Black fly trapping method** | HLC. 9 hours per day |
| **Sample type (heads / bodies / whole carcasses etc)** | Whole flies |
| **Max pool size** | 50 |

| **Rodriguez-Perez 2013** | |
| --- | --- |
| **Study Location** | Mexico. Three foci, Oaxaca, Southern Chiapas and Northern Chiapas. However, no parasitological data is available for Northern Chiapas. |
| **Timing of study** | 1998 - 2011 |
| **Epidemiological background** | Oaxaca communities: 89% hypo-endemic, 11% meso-endemic.   Southern Chiapas communities: 56% hypo-endemic, 37% meso-endemic, 7% hyper-endemic. |
| **History of MDA** | Semi-annual MDA with ivermectin since 1997 |
| **History of other interventions** | Nodulectomy campaigns since 1932 |
| **Study design** | Longitudinal observations |
| **Study objectives** | To observe the effect of ivermectin MDA on transmission and ultimately determine whether transmission has been interrupted. |
| **Sampling strategy (reference standard)** | Oaxaca: 4 sentinel villages (SVs), 21 Extra sentinel villages (ESVs)   Southern Chiapas: 6 SVs. 3 ESVs |
| **Frequency and timing of sampling (reference standard)** | Oaxaca:  ~2001 (exact date not described): skin snip (4 SVs) 2004: serology (4 SVs) 2007-08: serology (4 SVs and 21 ESVs), skin snips (4 SVs) and opthalmology (4 SVs)  Southern Chiapas: ~2001 (exact date not described): skin snip (2 SVs) Several other surveys conducted between 2001 and 2010 but not meetin g inclusion criteria for review (ie. serology screening with positives not confirmed by skin snip) |
| **Oncho diagnostic method** | Direct observation of skin snips, opthalmological examinations, and in some cases serology surveys in which no positives were detected |
| **Sampling strategy (index test)** | Two sites within each community (one in the community itself and one in a nearby coffee plantation). |
| **Frequency and timing of black fly sampling** | Feb - May 2001 |
| **Black fly trapping method** | HLC. One collector and one attractant working 6 hours per day |
| **Sample type (heads / bodies / whole carcasses etc)** | Heads and bodies separated. Bodies were screened first. If a positive body was detected, body-screening was stopped in that community and head-screening was undertaken |
| **Max pool size** | 50 |

| **Traore 2012** | |
| --- | --- |
| **Study Location** | Mali and Senegal. The three study areas are located along the River Bakoye in Mali, the River Gambia in Senegal, and the River Faleme on the border of the two countries |
| **Timing of study** | 2006 - 2010 |
| **Epidemiological background** | Hyperendemic prior to MDA. |
| **History of MDA** | Ivermection MDA started in 1988 in Mali and 1989 in Senegal |
| **History of other interventions** | No other interventions. |
| **Study design** | Longitudinal. |
| **Study objectives** | To find evidence of transmission suppression in the region after 14-17 years of MDA and give evidence on whether elimination is possible in the African continent |
| **Sampling strategy (reference standard)** | 40 - 57 villages in each of three study sites. All individuals over 1 year old invited to participate |
| **Frequency and timing of sampling (reference standard)** | Sample timepoints varied between study sites based on previous results showing progress towards elimination.   Phase 1: 2006 (IVM distributed after epi survey and before ento survey) Phase 2: 2007-08. Only ento surveys conducted. IVM stopped in test areas.  Phase 3: 2008-2010 Treatment stopped in all areas. Ento and epi surveys conducted twice (half the villages surveyed each time) |
| **Oncho diagnostic method** | Direct observation of skin snips |
| **Sampling strategy (index test)** | 4-6 fly catching points per study site |
| **Frequency and timing of black fly sampling** | Sample timepoints varied between study sites based on previous results showing progress towards elimination.   Phase 1: 2006 (IVM distributed after epi survey and before ento survey) Phase 2: 2007-08. Ento and epi surveys conducted. IVM stopped in test areas.  Phase 3: 2008-2010 Treatment stopped in all areas. Ento and epi surveys conducted twice |
| **Black fly trapping method** | HLC. 3-4 fly catchers working for 11 hours per day for each of 14 collection sites. |
| **Sample type (heads / bodies / whole carcasses etc)** | Body pools were analyzed first; if any of those pools were positive, all of the head pools analyzed to provide an estimate of the infectivity rate |
| **Max pool size** | 300 |

| **Zarroug 2016** | |
| --- | --- |
| **Study Location** | Sudan, Abu Hamed Focus |
| **Timing of study** | 2011 - 2015 |
| **Epidemiological background** | Mesoendemic; skin mf prevalence of 37% prior to treatment |
| **History of MDA** | Annual MDA with ivermectin implemented from 1998, increased to semi annual from 2006. Stopped in 2012 following evidence of interruption of transmission. |
| **History of other interventions** | None described |
| **Study design** | Longitudinal (assessment of transmission interruption and validation of elimination) |
| **Study objectives** | To verify elimination of onchocerciasis in the focus by WHO guidelines. |
| **Sampling strategy (reference standard)** | Sentinel villages in this study included the three communities (Kiji, ElBagair, and ElGeraif) with the highest original endemicity in the focus. In 2011, four additional communities in and around the Abu Hamed focus were assessed for parasitological indicators of the disease. These communities included three communities (Sharrari, Elfida, and Mukabrab) |
| **Frequency and timing of sampling (reference standard)** | 2007; 2011-12; 2014-15 |
| **Oncho diagnostic method** | Serology of school children to detect IgG antibodies to OV16 antigen confirmed by PCR of skin snip samples taken from positive cases |
| **Sampling strategy (index test)** | Four sample sites in and around sentinel villages |
| **Frequency and timing of black fly sampling** | December of 2010 to November of 2011 |
| **Black fly trapping method** | HLC. 5 days per month for 13 hours |
| **Sample type (heads / bodies / whole carcasses etc)** | Heads |
| **Max pool size** | 100 |
