# Supplementary material for: Evaluating the diagnostic test accuracy of molecular xenomonitoring methods for characterising the community burden of Onchocerciasis
Source: PLoS Negl Trop Dis. 2021 Oct 12;15(10):e0009812. doi: 10.1371/journal.pntd.0009812 (PMC8509893; doi:10.1371/journal.pntd.0009812)
Supplement: S4 Table — (DOCX) [file pntd.0009812.s004.docx]

**S4 Table: Explanations for assessments of methodological quality**

|  | Risk of Bias | | | | |  | Applicability domain | |
| --- | --- | --- | --- | --- | --- | --- | --- | --- |
|  | Index Test blinded | Reference standard blinded | Appropriate gap between surveys | Appropriate matching of sampled populations | Continuity of methodology over time |  | Applicability of participants for index | Applicability of participants for reference |
| **Botto 2016 [18]** | Low - not described but considered low risk due to study objectives | Low - not described but considered low risk due to study objectives | Unclear - not described in enough detail to allow a comparison | Unclear – as the human population is semi-nomadic, we cannot be sure of the extent to which flies collected in the areas where humans were surveyed were an accurate reflection of the fly populations the humans had been exposed to | N/A |  | Low - no concerns | Low - no concerns |
| **Convit 2013 [19]** | Low - not described but considered low risk due to study objectives | Low - not described but considered low risk due to study objectives | High - MDA was implemented between survey timepoints (and therefore no matched data was included in review) | Low - Matched villages | N/A |  | Low - no concerns | Low - no concerns |
| **Cruz-Ortiz 2012 [20]** | Low - not described but considered low risk due to study objectives | Low - not described but considered low risk due to study objectives | Low - time between entomological and parasitological surveys was less than 6 months | High risk - entomological surveys and parasitological surveys were not conducted in the same communities within the sampling area. | N/A |  | Low - no concerns | Unclear - Serological survey conducted in school children only |
| **Evans 2014 [22]** | Low - not described but considered low risk due to study objectives | Low - not described but considered low risk due to study objectives | Low - time between entomological and parasitological surveys was less than 6 months | Low - matched villages | N/A |  | Low - no concerns | Low - no concerns |
| **Guderian 1997 [23]** | Low - not described but considered low risk due to study objectives | Low - not described but considered low risk due to study objectives | Low - time between entomological and parasitological surveys was less than 6 months | Unclear - sampling sites for entomological survey not described | N/A |  | Low - no concerns | High - Skin snip survey participants were children under 5 only. |
| **Katabarwa 2020a [27]** | Low - not described but considered low risk due to study objectives | Low - not described but considered low risk due to study objectives | Low - time between entomological and parasitological surveys was less than 6 months | High - sampling sites for entomological surveys and parasitological surveys do not appear to have been matched | N/A |  | Low - no concerns | High - Skin snip survey participants were children aged 5-10 only |
| **Katabarwa 2020b [26]** | Low - not described but considered low risk due to study objectives | Low - not described but considered low risk due to study objectives | Low - time between entomological and parasitological surveys was less than 6 months | Low - sampling sites for entomological surveys were close to the surveyed villages | N/A |  | Low - no concerns | High - children under 10 years only |
| **Komlan 2018 [28]** | Low - not described but considered low risk due to study objectives | Low - not described but considered low risk due to study objectives | Low - time between entomological and parasitological surveys was less than 6 months | Low - sampling sites for entomological surveys were matched with surveyed villages | N/A |  | Low - no concerns | Low - no concerns |
| **Lindblade 2007 [29]** | Low - not described but considered low risk due to study objectives | Low - not described but considered low risk due to study objectives | Unclear - Timing of serological and opthalmological surveys not described | High - entomological sampling took place in 7 communities with highest vector densities, serological surveys covered all 70 communities and buffer areas. | N/A |  | Low - no concerns | Low - no concerns |
| **Nicholls 2018 [30]** | Low - not described but considered low risk due to study objectives | Low - not described but considered low risk due to study objectives | Low - time between entomological and parasitological surveys was less than 6 months | Low - sampling took place in the same village | N/A |  | Low - no concerns | Low - no concerns |
| **Richards 2015 [31]** | Low - not described but considered low risk due to study objectives | Low - not described but considered low risk due to study objectives | Low - time between entomological and parasitological surveys was less than 6 months | Unclear - 6 of the 9 sentinel villages were used in the sampling but it is not described which of the 6 and how many flies were collected in each | N/A |  | Low - no concerns | Low - no concerns |
| **Rodriguez-Perez 1999 [32, 33]** | Low - not described but considered low risk due to study objectives | Low - not described but considered low risk due to study objectives | Unclear - exact timing of parasitological survey not described. | Low - sampling was conducted in a single community | N/A |  | Low - no concerns | High - children under 18 only |
| **Rodriguez-Perez 2013 [34-36, 39]** | Low - not described but considered low risk due to study objectives | Low - not described but considered low risk due to study objectives. | Unclear - exact timing of surveys not always described. | Low - samples were conducted within target communities | N/A |  | Low - no concerns | Low - no concerns |
| **Traore 2012 [21, 37]** | Low - not described but considered low risk due to study objectives | Low - not described but considered low risk due to study objectives | Low - time between entomological and parasitological surveys was less than 6 months | High - Limited sample points in each study area (4-6 compared to 40 - 57 villages) | N/A |  | Low - no concerns | High - later surveys found difficulty achieving previous numbers as participants were reluctant to provide further skin samples. Six entire village refused to participate in the final survey. |
| **Zarroug 2016 [24, 25, 38]** | Low - not described but considered low risk due to study objectives | Low - not described but considered low risk due to study objectives | Low - time between entomological and parasitological surveys was less than 6 months | Unclear - entomological sampling took place in specific sampling sites in and around sentinel villages (but did not cover all sampled villages) | Low - No concerns |  | Low - no concerns | Low - no concerns |
