# Supplementary material for: Evaluating the diagnostic test accuracy of molecular xenomonitoring methods for characterising the community burden of Onchocerciasis
Source: PLoS Negl Trop Dis. 2021 Oct 12;15(10):e0009812. doi: 10.1371/journal.pntd.0009812 (PMC8509893; doi:10.1371/journal.pntd.0009812)
Supplement: S5 Table — (DOCX) [file pntd.0009812.s005.docx]

**S5 Table: Quantitative data extracted from included studies. Abbreviations: ND – Not Described; mf – Microfilaria; MX – Molecular Xenomonitoring; SL - Santiago Lalopa; ST - Santiago Teotlaxco; LE -** **La Esperanza ; SMLC -** **Santa María La Chichina.**

| **Study** | **Country** | **Sub-study Comparison** | **Human sample size** | **Black fly sample size** | **Human mf prevalence** | **MX rate** | **MX rate (head only)** |
| --- | --- | --- | --- | --- | --- | --- | --- |
| **Botto 2016 [18]** | Venezeula | Hasupiwei (2012-13) | ND | 8085 | 7% | 0 |  |
| **Botto 2016 [18]** | Venezeula | Pashopëka (2012-13) | ND | 6464 | 2% | 0 |  |
| **Botto 2016 [18]** | Venezeula | Koyowë (2012-13) | ND | 13117 | 7% | 0 |  |
| **Cruz-Ortiz 2012 [20]** | Guatemala | - | 3118 | 8252 | 0 | 0.00% |  |
| **Evans 2014 [22]** | Nigeria | Gurku | 354 | ND | 0 | 0 |  |
| **Evans 2014 [22]** | Nigeria | Arisi | 362 | ND | 0 | 0 |  |
| **Evans 2014 [22]** | Nigeria | Bakin Kogi Lemoro | 346 | ND | 0 | 0 |  |
| **Evans 2014 [22]** | Nigeria | Mafara | 410 | ND | 0 | 0 |  |
| **Evans 2014 [22]** | Nigeria | Kamwai | 351 | ND | 0.57% | 0 |  |
| **Evans 2014 [22]** | Nigeria | Godong | 374 | ND | 0 | 0 |  |
| **Guderian 1997 [23]** | Ecuador | 1990 | 225 | 10000 | 64.30% | 1.10% |  |
| **Guderian 1997 [23]** | Ecuador | 1996 | 233 | 10000 | 0.00% | 0.08% |  |
| **Katabarwa 2020a [27]** | Sudan and Ethiopia | Galabat | 3931 | 9148 | 0 | 0% |  |
| **Katabarwa 2020a [27]** | Sudan and Ethiopia | Metema | 4369 | 10744 | 0 | 0.0002 |  |
| **Katabarwa 2020a [27]** | Sudan and Ethiopia | West Armachicho | 1703 | 16839 | 0 | 0% |  |
| **Katabarwa 2020a [27]** | Sudan and Ethiopia | Alefa | 300 | 10862 | 0 | 0.0002 |  |
| **Katabarwa 2020a [27]** | Sudan and Ethiopia | Tach Armachicho | 300 | 110 | 0 | 0% |  |
| **Katabarwa 2020a [27]** | Sudan and Ethiopia | Quara | 300 | 27188 | 0 | 0.0001 |  |
| **Katabarwa 2020b [26]** | Uganda | - | 2953 | 854 | 0 | 0% |  |
| **Komlan 2018 [28]** | Togo | Oˆti/Pancery | 140 | 1025 | 2.9 | 0.20% |  |
| **Komlan 2018 [28]** | Togo | Ke´ran/Tchitchira | 146 | 875 | 10.3 | 1% |  |
| **Komlan 2018 [28]** | Togo | Moˆ/Baghan | 151 | 2575 | 8.6 | 0.10% |  |
| **Lindblade 2007 [29]** | Guatemala | - | 4127 | 11621 | 0 | 0 |  |
| **Nicholls 2018 [30]** | Colombia | 2001 | 143 | 5565 | 0 | 0.02% |  |
| **Nicholls 2018 [30]** | Colombia | 2004 | 232 | 10500 | 0.85% | 0.01% |  |
| **Rodriguez-Perez 1999 [32, 33]** | Mexico | - | 226 | 10550 | 13.00% | 0.009% |  |
| **Rodriguez-Perez 2013 [34-36, 39]** | Mexico | Oaxaca 2001 | ND | 21950 | 7% | 0.16% |  |
| **Rodriguez-Perez 2013 [34-36, 39]** | Mexico | Oaxaca 2004 - SL | 47 | 7600 | 0% | 0.023% | 0.000% |
| **Rodriguez-Perez 2013 [34-36, 39]** | Mexico | Oaxaca 2004 - ST | 28 | 2450 | 0 | 0.000% |  |
| **Rodriguez-Perez 2013 [34-36, 39]** | Mexico | Oaxaca 2004 - LE | 20 | 8450 | 0 | >0% (actual value ND) | 0.018% |
| **Rodriguez-Perez 2013 [34-36, 39]** | Mexico | Oaxaca 2004 - SMLC | 22 | 2950 | 0 | 0.000% |  |
| **Rodriguez-Perez 2013 [34-36, 39]** | Mexico | Oaxaca 2007-08 - SL | 395 | >10000 | 0 | 0.000% | 0.047% |
| **Rodriguez-Perez 2013 [34-36, 39]** | Mexico | Oaxaca 2007-08 - ST | 307 | >10000 | 0 | 0.000% | 0.739% |
| **Rodriguez-Perez 2013 [34-36, 39]** | Mexico | Oaxaca 2007-08 - LE | 112 | >10000 | 0 | 0.000% |  |
| **Rodriguez-Perez 2013 [34-36, 39]** | Mexico | Oaxaca 2007-08 - SMLC | 225 | >10000 | 0 | 0.000% |  |
| **Rodriguez-Perez 2013 [34-36, 39]** | Mexico | S. Chiapas 2001 | ND | 5300 | 16% | 0.71% |  |
| **Traore 2012 [21, 37]** | Mali and Senegal | R. Bakoye phase 3a | 3739 | 49800 | 0.05% | 0.000% |  |
| **Traore 2012 [21, 37]** | Mali and Senegal | R. Bakoye phase 3b | 3520 | 56700 | 0% | 0.000% |  |
| **Traore 2012 [21, 37]** | Mali and Senegal | R. Gambia phase 3a | 1561 | 83700 | 0.13% | 0.000% |  |
| **Traore 2012 [21, 37]** | Mali and Senegal | R. Gambia phase 3b | 1540 | 73200 | 0% | 0.000% |  |
| **Traore 2012 [21, 37]** | Mali and Senegal | R. Faleme phase 3a | 2301 | 122100 | 0.13% | 0.000% |  |
| **Traore 2012 [21, 37]** | Mali and Senegal | R. Faleme phase 3b | 4305 | 107100 | 0.07% | 0.002% |  |
| **Zarroug 2016 [24, 25, 38]** | Sudan | 2007 | 442 | 29401 | 0.50% | 0.01% |  |
| **Zarroug 2016 [24, 25, 38]** | Sudan | 2011-12 | 536 | 17359 | 0 | 0 |  |
| **Zarroug 2016 [24, 25, 38]** | Sudan | 2014-15 | 5266 | 19191 | 0 | 0 |  |
